# Small target detection of bearing surface defects based on improved YOLOv5s

**DOI:** 10.1371/journal.pone.0328892

**Published:** 2025-07-28

**Authors:** Haisong Xu, Xiaolin Shi, Han Zhang, Fan Yang, Yun Chen

**Affiliations:** 1 School of Mechanical Engineering and Automation, Liaoning University of Technology, Jinzhou, China; 2 Institute of Intelligent Manufacturing, School of Mechanical Engineering, Northwestern Polytechnical University, Xi’an, China; 3 College of Mechatronic Engineering, North Minzu University, Yinchuan, Ningxia, China; G H Raisoni College of Engineering and Management, Pune, INDIA

## Abstract

In the context of industrial automation, the accurate detection of small defects on bearing surfaces (dents, bruise, scratch) is crucial for the safe operation of equipment. However, traditional detection methods have problems such as insufficient feature extraction for small targets and sensitivity to background interference. Based on this, an improved YOLOv5s small target detection method ECN-YOLOv5s for bearing surface defects is proposed. First, an efficient channel attention mechanism (ECA) is inserted in the layer before the SPPF in the backbone network, which realizes efficient computation of channel attention and reduces redundant computation while decreasing the model complication. Second, using the Content-Aware ReAssembly of Features (CARAFE) module instead of the original nearest-neighbor up-sampling module achieves light weighting while allowing for better aggregation of contextual information within a larger sensory field, which effectively improves the diversity and effectiveness of the model. Finally, an original loss function is replaced with Normalized Wasserstein Distance (NWD) to decrease the susceptibility to small object positional deviations. Experimental results in our homemade dataset indicate that the mean average precision (mAP) of ECN-YOLOv5s for bearing defect identification is 92.7%. This is an improvement of 2.4% versus the former YOLOv5s model. Compared with some mainstream detection models, the modified model in this study also provides better effects. Thus, the model is able to meet the requirements for the detection of small defects in bearings in industry.

## Introduction

In recent years, in modern industrial production, bearings are indispensable components in the field of mechanical engineering, and are widely used in automobiles, lathes, aerospace, textiles, metallurgy, generators, wind turbines and a variety of industrial equipment [1–5]. The role of bearings is to support the rotating mechanical components and reduce friction, thus ensuring the stable operation of the mechanical system. Its quality will affect the reliability of the whole equipment. However, in the process of bearing manufacture and installation, it is inevitable to be affected by processing, assembly methods and tiny foreign objects, which lead to some defects on the surface of some bearings. Common types of defects are dent defects, bruise defects, and scratch defects. Such defects will have an impact on bearing quality and function, leading to failures such as unbalance of rotating parts and vibration of the equipment, thus affecting the stability and reliability of the equipment [6]. Therefore, the surface of the bearings must be subjected to strict quality inspection before leaving the factory to control its production quality.

At present, domestic bearing manufacturers have basically realized automated production and automated assembly, but the quality inspection of bearings still relies on visual inspection by experienced workers [7]. The method of manual visual inspection is slow and inefficient, and cannot satisfy the demand of automated industrial operation. Therefore, the research of fast, accurate and non-destructive detection of defects on the product surface is of great significance. And the target detection algorithm has a strong detection capability. Target detection algorithms can be categorized into two-stage target detection algorithms and single-stage target detection algorithms [8,9]. The two-stage target detection algorithm [10] has been suffering from unsolved issues like complex model and slow identification rate. The one-stage target detection algorithm [11], on the other hand, simplifies this model and is able to maintain a fairly high identification precision while improving the identification rate. Considering the multifaceted advantages of single-stage target detection, and YOLO [12] series as the most widely used class of single-stage target detection models. Therefore, in this paper, based on YOLO’s powerful ability in image detection, we collect the image information of the bearing, create our own dataset, and on the basis of this, train and optimize the parameters by YOLOv5, to further improve the efficiency as well as the accuracy of image classification.

For this reason, this paper proposes an improved YOLOv5s method for small target detection of bearing surface defects. The method aims to optimize the problems of high false detection rate, high misdetection rate and low efficiency in identifying bearing surface defects. The detailed contributions of this article are listed below:

(1) Data enhancement: In this article, a portion of the dataset will be self-created and then data enhancement will be performed by cropping, rotating, panning, mirroring, adding noise and other operations to the image. These techniques can improve the diversity and accuracy of the dataset to enhance the reliability and feasibility of image recognition.
(2) Attention mechanisms: In order to reduce the problems of complex texture features and a lot of interfering information in bearing surface defect images. Reduce the phenomenon that the extracted feature map has a lot of redundant information in the dimensions of space and channel. In this paper, ECA attention mechanism is introduced to enhance the adaptability and the generalization ability of convolutional neural network.
(3) Upsampling algorithm: Due to the fact that the bearing defect morphology is mostly small, which makes it difficult for us to recognize it. So the CARAFE upsampling algorithm is introduced, which captures richer scene structure and details and improves the model’s recognition ability.
(4) Loss function: This paper changes the original loss function of YOLOv5s to NWD to reduce the susceptibility to small target location deviation. Thus, it has a better effect on the recognition of small targets.

The remainder of the article is organized in the following way: the section “Related Work” describes the related work. The section “Methodology” outlines the YOLOv5s network structure as well as describes in detail the improved ECN-YOLOv5s network of the paper. The results of the experiments are analyzed and discussed in Part “Experimental Results and Analysis”. Conclusions and future perspectives are given in Part “Conclusions”.

## Related work

### Status of surface defects in industrial products

Since convolutional neural networks have strong capability of extracting features, many researchers have begun to adopt deep learning methods to address the flaw detection problem on the surface of industrial products. Lei et al. [13] presented a primitive defect detection approach: the Segmented Embedded Rapid Defect Detection for Surface Defects (SERDD). The method achieves a two-way fusion of picture processing and flaw detection. It is able to effectively and precisely check surface defects like dents, nicks, gouges oily stains, and dimensional anomalies. Kankar et al. [14] applied Artificial Neural Networks (ANN) and Support Vector Machines (SVM) for fault diagnosis of ball bearings, which are better at recognizing bearing cracks, corrosion, etc. Gu et al. [15] proposed a machine vision based method for automatic detection and recognition of bearing surface defects. The completeness and accuracy of bearing surface defects recognition were improved by improving Canny’s algorithm and Otu’s algorithm. Kim et al. [16] combined data preprocessing methods and hyperparametric methods in deep learning to study the impact on ball bearing fault detection accuracy and achieved high accuracy in complex situations. Zheng et al. [17] suggested an improved YOLOv3 model including Bottleneck Attention Network (BNA-Net), Attention Prediction Sub-Network Module, Defect Location Sub-Network Module, and Output Feature Branches of Large Size, which solved the problem of insensitivity to large targets in bearings. Bapir and Aydin [18] proposed a one-dimensional convolutional neural network for diagnosis and identification of early bearing faults based on variational modal decomposition (VMD). Udmale et al. [19] presented an intellectual diagnosis method of fault classification based on deep learning Kurtogram and Sequence Model (SM) and achieved a good fault classification accuracy. Jiang et al. [20] integrated bispectral analysis with a modified integrated ensemble empirical modal decomposition (EEMD) to effectively reduce Gaussian and non-Gaussian noise in the bearing defect test set.

The above research results show that deep learning object inspection algorithms are very widely used in the mechanical manufacturing industry, and also have good results in recognizing defects in mechanical parts. However, there are still some shortcomings in the recognition of small defects with overlapping random locations.

### Status of YOLO target detection technology

The YOLO series, as the most widely used class of single-stage target detection models, are able to not only provide high inspection precision, but at the same time to detect the location information and category information of the target object, and to realize end-to-end training. so the detection speed is faster. REDMON et al. [21] first proposed the YOLO algorithm in 2015, and since then the algorithm series keeps on iterating and is extensively utilized in various fields. Kou et al. [22] proposed an end-to-end flaw inspection module on the basis of YOLOv3 to recognize the surface defects of steel strips, and improved the model detection performance by adding an anchor point feature selection mechanism and introducing a dense convolutional block. Li et al. [23] improved YOLOv4 and proposed a solution to detect defects in wire and arc additive manufacturing (WAAM) to achieve accurate detection of WAAM. Zhou et al. [24] proposed a method based on the defect detection model YOLOv8-2d and balanced generative adversarial network (BAGAN). It optimizes the problems of lower inspection accuracy, slower inspection rate, and unbalanced flaw specimens in the detection of surface defects on non-standard parts. Shuai et al. [25] proposed a (SF)-YOLO to optimize the algorithm for the extracted important regions of the gears in order to accomplish the automatic detection of gear defects. Qu et al. [26] presented an improved single-stage object detector based on the YOLOv5 method to improve its leakage and misdetection problems for large objects and enhance the recognition and detective capabilities of the model. Xiao et al. [27] put forward the YOLOv5-TB model by combining Transformer and Bi-FPN with YOLOv5. The model is used for galvanized steel defect detection, and it has good detection effect on zinc flower defects on galvanized steel surface.

## Methodology

### ECN-YOLOv5s

**Fig 1:**
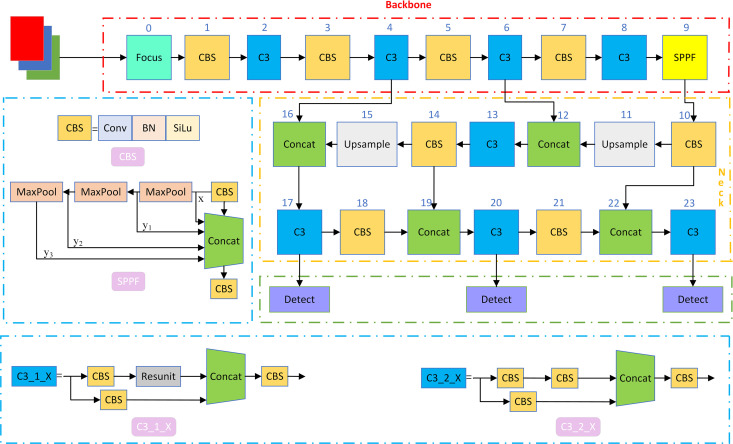
YOLOv5s general structure diagram.

The YOLOv5 network can be divided into 4 parts, which are the inputs, the backbone network, the neck network and the detection layer. There are five versions of the YOLOv5 target inspection module: YOLOv5n, YOLOv5s, YOLOv5m, YOLOv5l, and YOLOv5x. Considering the need for bearing defect detection algorithms to satisfy the demands of real-time as well as lightweight detection, although YOLOv5n is smaller than YOLOv5s and more lightweight, it is only suitable for scenarios with very low requirements on hardware resources. Therefore, in this paper, YOLOv5s is selected as the baseline model for target inspection. The general framework of YOLOv5s algorithm is shown in Fig 1.

**Fig 2:**
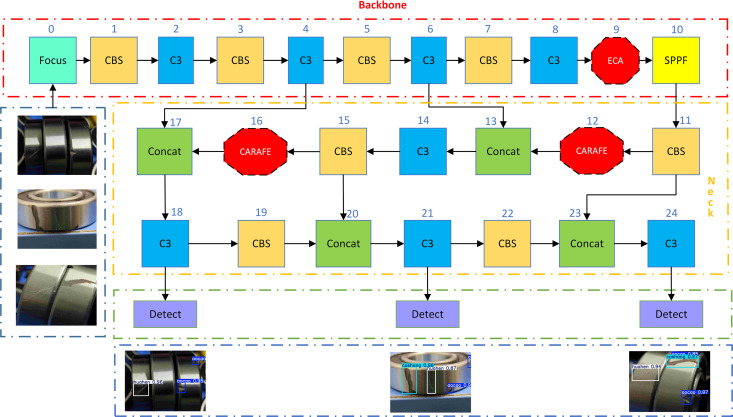
ECN-YOLOv5s structure diagram.

As a better way to realize the recognition of bearing surface defects, the original model of YOLOv5s is improved in this paper. The improved model is shown in Fig 2. First, an attention mechanism, the ECA, is implemented in Backbone to reduce the complexity and redundancy of image recognition by allowing the model to focus more effectively on task-pertinent information through adaptive channel significance adjustment. Achieve efficient recognition of images. Second, the up-sampling module in Neck was replaced with CARAFE, which is capable of aggregating contextual information over a larger sensory field, which helps to capture richer scene structure and detail. Finally, the loss function is changed to NWD, the bounding frame is modeled with a two-dimensional Gaussian distribution, and the similarity between the predicted and actual objects is calculated using the Gaussian distribution and the distributions corresponding to them. It also improves the recognition of overlapping small targets by better recognizing them.

### Efficient channel attention mechanism

**Fig 3:**
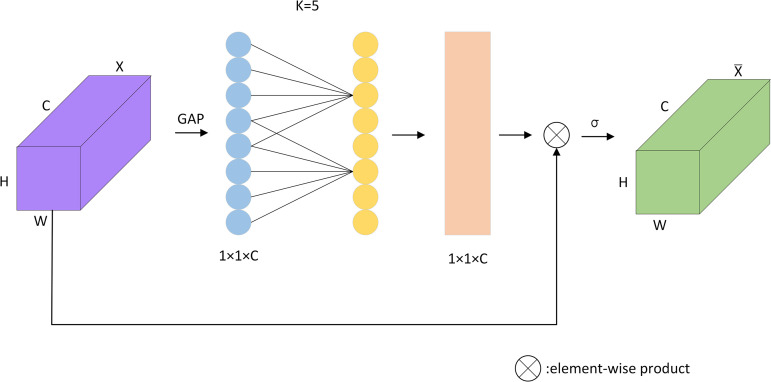
ECA structural model diagram.

In bearing defect detection tasks, low-contrast defects are often characterized as weak and difficult to recognize, which poses a significant challenge. The low contrast between these defects and the surrounding background results in them not being visible enough in the image and can easily be overlooked or misjudged. However, the introduction of the ECA module provides an effective solution to this problem. One kind of attention mechanism utilized in image recognition tasks is the ECA [28] attention mechanism. It is inspired by the SE [29] attention mechanism, which dynamically modifies the priority of channels in a CNN model to improve feature representation. The goal of the channel attention mechanism used in CNN models is to improve the networks’ capacity to recognize connections between channels. Each channel’s feature map is typically regarded as independent in conventional CNNs, and each channel is given equal weight. In real-world images, however, various channels can display different levels of relation and importance. This issue is addressed by the ECA attention mechanism, which uses adaptive channel priority adjustment to help the network concentrate more effectively on task-relevant input. The method has been shown to be highly effective in enhancing the capacity of the model to represent key channel characteristics. Compared to the traditional attention mechanism, the ECA attention mechanism concentrates more on the correlation among channels than the relationship of sequence data. It computes the weights of each channel by using a one-dimensional convolution operation and then applies these weights to the corresponding feature drawings. The channel attention weights are multiplied to scale the feature drawings for each channel. This allows important channels to be strengthened and unimportant channels to be suppressed. The final obtained feature map is used for subsequent tasks as shown in Fig 3. Such a design not only decreases the total amount of model parameters but also reduces the computational cost.

Specifically, giving each channel a weight is the fundamental idea underlying ECA channel focus. In order to accomplish this, a learnable single-dimensional convolutional layer is introduced, which pools the global average of each channel’s feature maps to aggregate the global description of each channel. A convolution kernel is used to apply a single-dimensional convolution process for each input channel. A sigmoid function then converts the convolution result into weight values between 0 and 1 to reflect the attention weights. The following equation (1) is the ECA formula used to generate the channel weights by a single-dimensional convolution with size K:


$$\omega =\sigma \left(C1D_{k}\left(y\right)\right)$$ (1)

where σ stands for a Sigmoid activation function, C1D for a single-dimensional convolution, and y for a channel; the greater the channel dimension, the wider the spectrum of local cross-channel connections. equation (2) below provides the mapping of the channel dimensions C and K:


C=ϕ(K)≈exp(γ×K−b) (2)

The kernel size K is adaptively determined given the channel dimension C as in equation (3):


$$K=\psi \left(C\right)=|\frac{{\log_{2}}^{\left(C\right)}}{\gamma }+{\frac{b}{\gamma }|}_{odd}$$ (3)

where the two hyperparameters, γ and b, are utilized to modify the mapping function’s shape and, consequently, the weight of attention. To maximize the model’s performance, γ and b are typically set to particular values, such as b = 1 and γ = 2.${\left|\right|}_{odd}$denotes the closest odd number of K to the function after taking its absolute value.

### CARAFE

The sampling method in YOLOv5 generally utilizes traditional bilinear interpolation for feature map upsampling, which is relatively simple. It sizes the feature map to the desired target resolution by interpolating the pixels in the feature drawing to increase the feeler field and localization accuracy in the model at high resolution. But the method does not utilize the feature map’s semantic information. As a pervasive upsampling, it has a small perceptual domain and tends to ignore the information of the features as a whole.

One of the major challenges in the inspection of bearing defects is that the bearing targets are too small. As a result, defects are more difficult to detect, so this requires high-resolution feature maps to achieve accurate detection of bearing defects. Therefore, CARAFE [30] is used in this paper to replace the original nearest neighbor upsampling technique in YOLOv5. The CARAFE module can effectively enhance the feature details of small targets, enabling the model to recognize the boundaries and shapes of small targets more clearly. By retaining more feature details, the CARAFE module helps the model to better distinguish small targets in complex backgrounds, which significantly improves the model’s localization accuracy of small targets.

**Fig 4:**
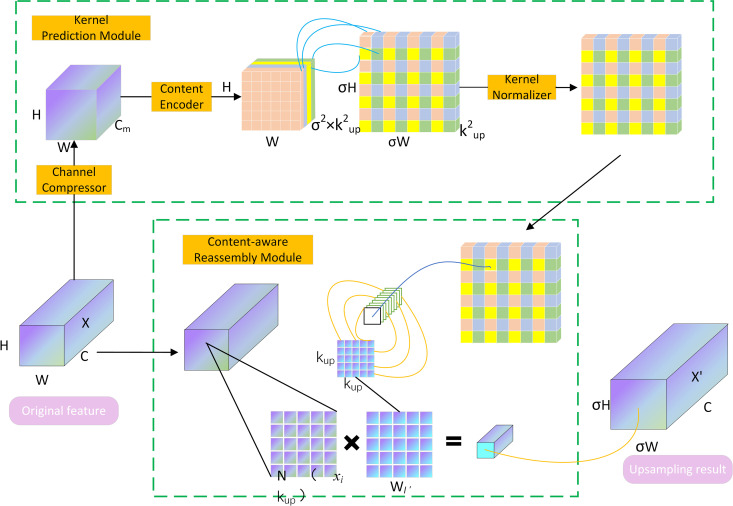
CARAFE general structure.


Unlike the traditional bilinear interpolation nearest-neighbor upsampling technique, this sampling method is able to aggregate contextual information over a larger sensory field, which helps to capture richer scene structure and details. Additionally, CARAFE eschews the traditional approach of using a normal kernel for all specimens (e.g., inverse convolution) and instead implements instance-specific content adaptive processing. This means that it is able to dynamically generate adaptable kernels that flexibly adjust to the input content. Moreover, the method introduces minimal computational overhead and is easy to integrate into neural network architectures without significantly increasing model complexity or slowing down inference. The CARAFE module, as demonstrated in Fig 4, consists of two primary parts, the Kernel Prediction Module and the Content-aware Reassembly Module. Predicting the up-sampled kernel for every target location is the primary responsibility of the kernel prediction module. Since these upsampling kernels are dynamically generated according to the content of the supplied feature drawing, they may vary at different locations. The main task of the content-aware reassembly module is to reorganize the supplied feature drawing using the upsampling kernel predicted by the kernel forecast module to generate the up-sampled feature drawing. Specifically, As shown in equation (4), given the input feature map$X\in R^{H\times W\times C}$,the input H × W × C feature map is compressed using feature map channel compression in the upsampling kernel prediction section into H × W × Cm using 1 × 1 convolution, which effectively reduces the computation.

$$X_{m}=Conv_{1\times 1}(X),\;X_{m}\in R^{H\times W\times C_{m}}$$ (4)

With an upsampling rate of σ and an upsampling size of kup × kup, content encoding and upsampling of kernel predictions are realized by changing the number of channels for content encoding from Cm to $\sigma^{2}\times k_{{up}^{2}}$ through convolutional operations. As shown in equation (5):


$$K=Conv_{k_{encoder}\times k_{encoder}}(X_{m}),\;K\in R^{\sigma^{2}\times k_{up}^{2}\times H\times W}$$ (5)

where $k_{encoder}$ is the convolutional kernel size of the content encoder.

After then, the channel’s dimension of space is expanded. The feature maps are sent to the feature reorganization stage once the prediction results have undergone SoftMax normalization. And the upsampling result is obtained by multiplying the features on every single layer of the map of characteristics by the projected upsampling kernel. As indicated in equation (6):


$$X_{\left(i,j\right)}^{\prime }=\sum_{n=-r}^{r}\sum_{m=-r}^{r}K_{\left(i,j,n,m\right)}\cdot X_{\left(i+n,j+m\right)}$$ (6)

where $X_{\left(i,j\right)}^{\prime }$ is the value of the output feature map at position (i,j), $K_{\left(i,j,n,m\right)}$ is the value of the kernel at position (i,j,n,m), and $X_{\left(i+n,j+m\right)}$ is the value of the input feature map at position (i+n,j+m).

### NWD loss function

**Fig 5:**
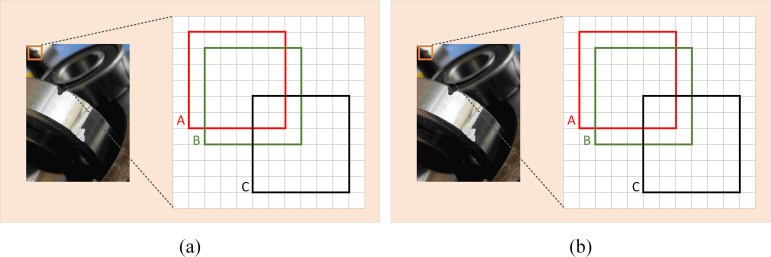
Comparison of IoU sensitivity. **(a)** Minor defects. **(b)** Normal defects. Box A denotes the ground truth bounding box, and boxes B and C denote the predicted bounding boxes with 1-pixel and 4-pixel diagonal deviations, respectively.


The bounding box loss of YOLOv5s defaults to CIoU; however it is extremely sensitive to small target position changes in the bounding box [31]. In Fig 5, for a tiny defect of 6 × 6 pixels, as shown in equation (7) and equation (8), a small positional deviation leads to a significant IoU change (from 0.53 to 0.06). Whereas, when the change in positional deviation is the same, for a normal-sized defect of 36 × 36 pixels, as shown in equations (9) and equation (10), the IoU change is small (only decreasing from 0.90 to 0.65).

$$IoU=\frac{\left|A\cap B\right|}{\left|A\cup B\right|}=0.53$$ (7)

$$IoU=\frac{\left|A\cap C\right|}{\left|A\cup C\right|}=0.06$$ (8)

$$IoU=\frac{\left|A\cap B\right|}{\left|A\cup B\right|}=0.90$$ (9)

$$IoU=\frac{\left|A\cap C\right|}{\left|A\cup C\right|}=0.65$$ (10)

Moreover, in bearing defect detection, the defect targets are generally small and not strictly rectangular, which may lead to unsteadiness of the loss function. In order to better address the issue, this paper introduces the NWD loss function [32], which models the enclosing frame as a 2-D Gaussian distribution, using the Gaussian distribution and the distributions corresponding to the predicted and actual objects to compute the similarity between them. It also improves the recognition of overlapping small targets better. For the horizontal enclosing frame R=(cx, cy, w, h), where cx, cy represent the center point coordinates, and w and h represent the enclosing frame’s width and height. One way to express its elliptic equation is as:


$$\frac{{\left(x-\mu_{x}\right)}^{2}}{\sigma_{x}^{2}}+\frac{{\left(y-\mu_{y}\right)}^{2}}{\sigma_{y}^{2}}=1$$ (11)

where $\sigma_{x}$ and $\sigma_{y}$ denote the semi-axis length along the x-axis and y-axis and ($\mu_{x}$,$\mu_{y}$) are the ellipse’s coordinates of center. Thus $\mu_{x}=cx$, $\mu_{y}=cy$, $\sigma_{x}=\frac{w}{2}$, $\sigma_{y}=\frac{h}{2}$.

The 2-D Gaussian distribution’s probability density function can be written as follows:


$$f\left(x|\mu ,\sum \right)=\frac{\exp\left(-\frac{1}{2}{\left(x-\mu \right)}^{T}\sum^{-1}\left(x-\mu \right)\right)}{2\pi {\left|\sum \right|}^{\frac{1}{2}}}$$ (12)

where x denotes the Gaussian distribution coordinates (x, y), μ stands for the mean vector, and Σ represents the covariance matrix. A 2-D Gaussian distribution N(μ,Σ) can be used to simulate the horizontal bounding box R=(cx, cy, w, h), where:


$$\mu =\left[\begin{array}{c} cx\\ cy \end{array}\right]\sum =[\begin{array}{cc} \frac{\omega^{2}}{4} & 0\\ 0 & \frac{\omega^{2}}{4} \end{array}]$$ (13)

The second-order Wasserstein distance between two 2-D Gaussian distributions μ1=N(m1,Σ1),μ2=N(m2,Σ2) can be defined as follows:


W22(μ1,μ2)=‖m1−m2‖22+Tr(∑1+∑2−2(∑2\raise0.7ex1/12\nulldelimiterspace\lower0.7ex2∑1∑2\raise0.7ex1/12\nulldelimiterspace\lower0.7ex2)\raise0.7ex1/12\nulldelimiterspace\lower0.7ex2) (14)

The above equation can be simplified as:


W22(μ1,μ2)=‖m1−m2‖22+‖∑1\raise0.7ex1/12\nulldelimiterspace\lower0.7ex2−∑2\raise0.7ex1/12\nulldelimiterspace\lower0.7ex2‖F2 (15)

where ${\left|||\right|}_{F}$ stands for the Frobenius norm.

By the boundary boxes A(${cx}_{a}$,${cy}_{a}$,$\omega_{a}$,$h_{a}$) and B(${cx}_{b}$,${cy}_{b}$,$\omega_{b}$,$h_{b}$) for the Gaussian distributions Na and Nb modeled, they can be expressed according to the above equation as:


$$W_{2}^{2}\left(N_{a},N_{b}\right)={\left\|\left({\left[cx_{a},cy_{a},\frac{\omega_{a}}{2},\frac{h_{a}}{2}\right]}^{T},{\left[cx_{b},cy_{b},\frac{\omega_{b}}{2},\frac{h_{b}}{2}\right]}^{T}\right)\right\|}_{2}^{2}$$ (16)

However, as the distance measure cannot be used straightway for measures of comparability, equation (17) illustrates how the distance measure is normalized in exponential form to produce the Normalized Wasserstein Distance, called NWD. where the mean absolute measurement of the dataset target is represented by the constant C. The bearing defect dataset in this paper contains defects of different sizes. By calculating the dimensions of all defect targets and averaging them, C = 0.8 is obtained.


$$NWD\left(N_{a},N_{b}\right)=\exp\left(-\frac{\sqrt{W_{2}^{2}\left(N_{a},N_{b}\right)}}{C}\right)$$ (17)

In this study, we control the respective contributions of IoU and NWD in the overall dropped frames and set the weight value of each to 0.5, which guarantees that the contributions of IoU and NWD to the overall dropped frames are identical.

## Experimental results and analysis

### Image recognition of bearing defects

#### Data acquisition.

A target detection job for bearing flaws is presented in this research, but there are fewer public datasets about bearing defects, and the sample diversity and size of the existing datasets are difficult to meet the needs of deep learning model training. So this paper makes its own dataset. The collection of bearing defect sample data is mainly obtained by taking photographs of the physical bearing. An industrial camera is used to photograph the defective bearing in order to obtain a part of the defective image. Not all images meet the requirements of the dataset due to the possibility of uneven illumination, background interference, and inconsistent resolution in directly captured images. Therefore, the collected data samples first need to undergo a strict pre-processing operation. First, blurry, overexposed, or underexposed images are rejected by manual screening. Second, the image is cropped and scaled to ensure that the target defective region is located in the center of the image and occupies an appropriate proportion. Finally, the image resolution is uniformly adjusted to facilitate subsequent model processing. To further enhance the size and diversity of the dataset, data enhancement was performed using operations such as rotation, panning, mirroring, and adding noise.

#### Part dataset creation.

**Fig 6:**
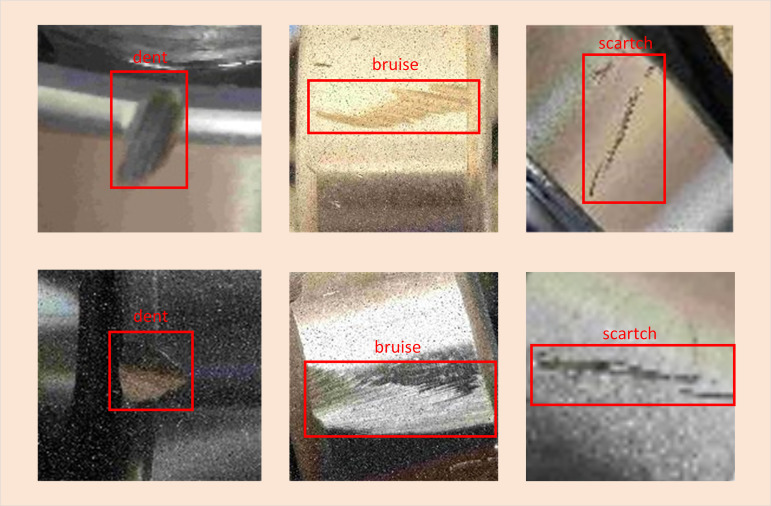
Images of different defect categories. “dent” means a localized depression on the bearing surface; “bruise” is the appearance of scrapes on the bearing surface due to impact or collision of external forces; “scratch” refers to the linear or curved shape of the traces formed on the bearing surface.

Supervised training is applied to train the recognition of a self-made dataset of bearing defect images. The dataset includes three categories of bearing’s dent defects, bruise defects, and scratch defects, totaling 5824 bearing defect images. The image size is 554 × 416 pixels and 312 × 416 pixels, and in this paper, defects smaller than 32 × 32 pixels are defined as “small target defects”. The number of the three types of defects and their proportion to the overall defects are shown in Table 1. It can be seen that the proportion of each defect in the overall number of defects is relatively close to ensure the balance of defect categories. In this paper, defective images are labeled using the LabelImg picture annotation tool, and all images are randomly assigned to training, validation, and test sets. With an 8:1:1 distribution ratio, there are 4660 photos in the training set, 582 images in the validation set, and 582 images in the test set. The initial learning efficiency of the weights adopted in network training is 0.001, and the attenuation coefficient is 0.0005. Each defective category’s magnified images are displayed in Fig 6.

**Table 1:** Defect category statistics.

Defect category	Number of defects	Percentage of total
Dent	6173	38%
Bruise	5462	33%
Scratch	4796	29%

### Laboratory equipment

Python is selected as the language of programming, the experiment makes use of the Pycharm integrated development environment, and it is executed on an effective platform to guarantee its seamless operation. Table 2 displays the particular experimental setup and associated parameters:

**Table 2:** Experimental environment and parameters.

Experimental Equipment	Configuration
Operating system	Windows 11
CPU	i9-13900KF
GPU	NVIDIA GeForce RTX4080
CUDA	12.1
Framework	Python3.8.19 Pytorch2.3.1
Platform	Pycharm 2023

### Assessment of indicators

In this paper, four evaluation metrics are used in order to validate the improved model detection capability. Precision denotes the proportion of samples that are truly positively classified out of the samples identified as positively classified by the model. Recall denotes the ratio of the amount of samples properly identified as positively classified to the total amount of positively classified. The average of precision values across several categories and recall levels is known as mean Average Precision(mAP). FPS stands for frames per second, which measures how many photos a model can process in a second on a specific piece of hardware. In order to achieve real-time detection, this statistic is crucial. The formula for calculating each metric is given below:


$$P=\frac{TP}{TP+FP}$$ (18)

$$R=\frac{TP}{TP+FN}$$ (19)

$$AP=\int_{0}^{1}P(R)dR$$ (20)

$$mAP=\frac{\sum {\mathrm{\backslash nolimits}}_{n=1}^{M}AP_{n}}{M}$$ (21)

True Positive (TP) in this context refers to the model’s accurate prediction that samples that fall into the positive category will also fall into that category. When a model predicts samples that belong in the negative group to be in the positive category, this is known as a false positive (FP).When a model predicts samples that belong in the positive category to be in the negative group, this is known as a false negative (FN).

### Analysis of experimental results

According to the algorithm suggested in this paper, a bearing surface defect recognition model, ECN-YOLOv5s, based on improved YOLOv5s is constructed.

**Fig 7:**
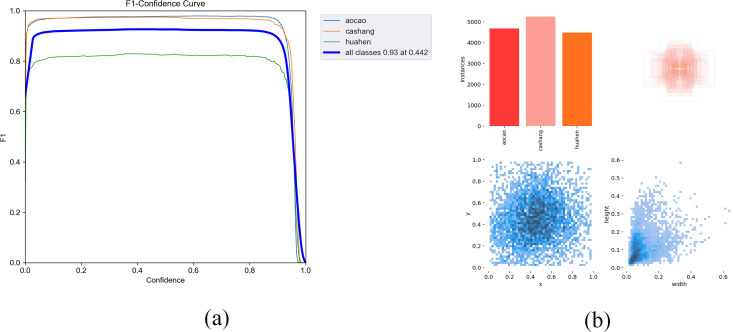
(a) F1 score curve; (b) Distribution of dataset label visualizations.

The F1 score is the reconciled average of precision and recall. Fig 7a demonstrates the curve of F1 score, and it can be seen that the F1 score reaches 0.93, which proves that the method proposed in this paper classifies better and enhances the accuracy and comprehensiveness of the prediction of positive samples.

Fig 7b illustrates the dataset label visualization distribution graph, from which one can understand the object categories of the dataset, the distribution of object center of mass locations, the location of horizontal and vertical coordinate centers of mass, and the distribution of object sizes. This provides valuable insights for assessing the usability and confidence of the dataset.

**Fig 8:**
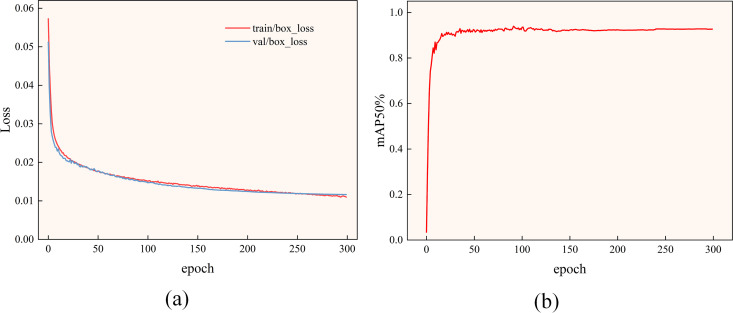
(a) Training and validation loss curves; (b) mAP50 curve.

Fig 8a shows the box_loss variation curves for training and validation of this improved model; the curves show that the loss decreases significantly during initial training. After about 100 rounds of iterations, the curves become slow to decrease and the training and validation loss curves level off. This shows that the model’s localization performance is improving in both training and validation data without significant overfitting and underfitting. It also learns more about the patterns in the training data.

Fig 8b shows the growing trend of mAP50 for ECN-YOLOv5s. After about 100 rounds of training, the mAP50 value of the improved model stabilizes and the average accuracy exceeds 92%. It shows that the model has sufficiently learned the effective information from the training data. In the later stages of training as the number of training rounds increases and some external factors interfere, the value of mAP50 may fluctuate slightly, but the overall level remains high. At this point it can also be concluded that the model has completed training.

**Fig 9:**
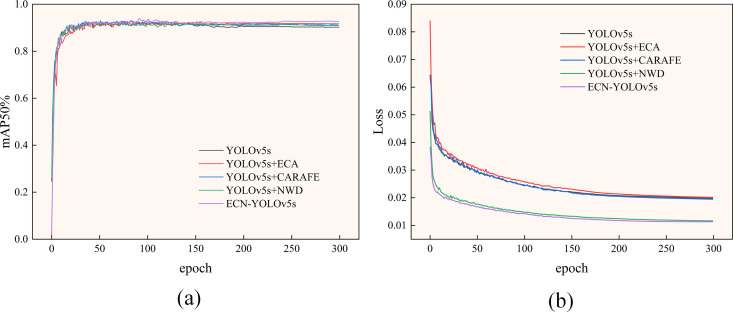
(a) Comparison curves of mAP50 for each model; (b) Comparison curves of loss values for each model.

Fig 9a illustrates the mAP50 comparison curves for YOLOv5s, YOLOv5s+ECA, YOLOv5s+CARAFE, YOLOv5s+NWD and ECN-YOLOv5s. After about 100 rounds of iterations, the mAP50 curves of several models are close to leveling off. Among them, the YOLOv5s model achieves an average precision of about 90%; the YOLOv5s+ECA model achieves an average precision of about 91%; the YOLOv5s+CARAFE model achieves an average precision of about 91%; and the YOLOv5s+NWD model achieves an average precision of about 92%. It is observed that our final improved model based on YOLOv5s has the highest average accuracy. The average accuracy values are improved by 2.4%, 1.5%, 1.6%, and 1% relative to the YOLOv5s, YOLOv5s+ECA, YOLOv5s+CARAFE, and YOLOv5s+NWD models, respectively, which show the best performance.

Fig 9b shows the comparison curves of training loss for several models. It is clear that ECN-YOLOv5s reaches the convergence state more quickly and has less loss. The YOLOv5s model exhibits significant instability in the initial phase, whereas our final improved model exhibits a smoother training loss profile and a training loss that is approximately 67% lower than the YOLOv5s model. The results show that the presented approach minimizes the loss and effectively solves the overfitting. Thus, the enhanced model in this paper has a more stable and effective training procedure.

### Comparison experiment

#### Comparative experiments with different attentional mechanisms.

In order to verify the advantages of the ECA attention mechanism chosen in this paper, the three attention mechanisms, CA, CBAM and SimAM, are added to the YOLOv5s model and compared with the YOLOv5s model with the ECA attention mechanism added. The experimental results are shown in Table 3. The results in the table show that the mAP50 value reached 91.2% after adding the ECA attention mechanism in YOLOv5s. Compared to adding the three attentional mechanisms, CA, CBAM, and SimAM, in YOLOv5s, the mAP50 increased by 0.4%, 0.4%, and 2%, respectively. The experimental results indicate that the ECA attention mechanism shows the best results in the recognition of bearing surface defects in this paper.

**Table 3:** Performance comparison of different attention mechanisms.

Attention mechanism	P(%)	R(%)	mAP50%
CA-YOLOv5s	92.9	90.9	90.8
CBAM-YOLOv5s	93.9	90	90.8
SimAM-YOLOv5s	94.1	87.1	89.2
ECA-YOLOv5s	93.3	90.8	91.2

#### Comparative experiments with different loss functions.

**Fig 10:**
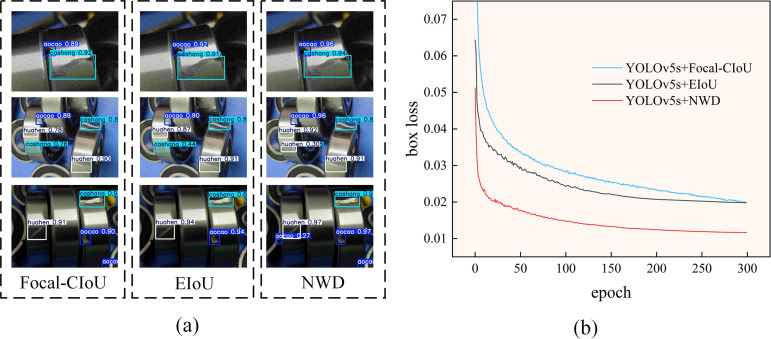
(a) Comparison of different loss functions for small target defect detection; (b) Comparison of localization loss for small target defect detection with different loss functions.

In order to verify the effectiveness of the NWD loss function, this study conducted a comparison experiment using the SIoU, EIoU, GIoU loss functions, and the NWD loss function. The experimental results are shown in Table 4, and the following conclusions can be drawn by analyzing the results in Table 4.The NWD loss function has the best performance during training. Compared to the other four loss functions, YOLOv5s has the highest mAP50 value of 91.7% when using the NWD loss function as the bounding box regression loss. Therefore, the NWD loss function exhibits the best performance in this comparison experiment with higher accuracy and robustness. In addition, in order to verify the advantage of NWD in small target detection, some detection datasets with small target defects are selected, and the NWD loss function is compared with other common small target optimization loss functions (Focal-CIoU and EIoU) in the comparison experiments. As shown in Fig 10(a), it can be visualized that NWD is more accurate in identifying small target defects. Fig 10(b) demonstrates the error comparison between the prediction box and the calibration box of several models, and it can be seen that the localization loss of the model incorporating NWD for small targets is smaller than that of several other models, which also verifies the advantage of NWD in small target detection.

**Table 4:** Performance comparison of different loss functions.

Loss function	P(%)	R(%)	mAP50%
SIoU-YOLOv5s	94.1	91.4	91.1
EIoU-YOLOv5s	94.6	90.8	91.5
GIoU-YOLOv5s	94.6	90.3	91.1
NWD-YOLOv5s	93.8	90.5	91.7

#### Comparative experiments of different lightweighting models.

In order to verify that the improved model proposed in this paper can show significant advantages in the task of bearing surface defect detection while maintaining lightweight and real-time performance. In this paper, the improved model is compared with the original model, YOLOv8n and YOLOv10s, which are several lightweight models, and the experimental results are shown in the Table 5. The information in the table shows that the improved model performs well in the bearing defect detection task. It outperforms the other lightweight models in terms of detection accuracy, and at the same time, the improved model maintains its lightweight nature, which makes it suitable for deployment in resource-constrained industrial environments.

**Table 5:** Performance comparison of different lightweighting models.

Model	Parameters (10^6^M)	FLOPs(G)	mAP50%	FPS
YOLOv5s	7.02	15.8	90.3	117
YOLOv8n	3.01	8.2	91.9	204
YOLOv10s	8.04	28.6	90.2	189
ECN-YOLOv5s	7.36	16.6	92.7	120

#### Performance analysis of each algorithm.

**Fig 11:**
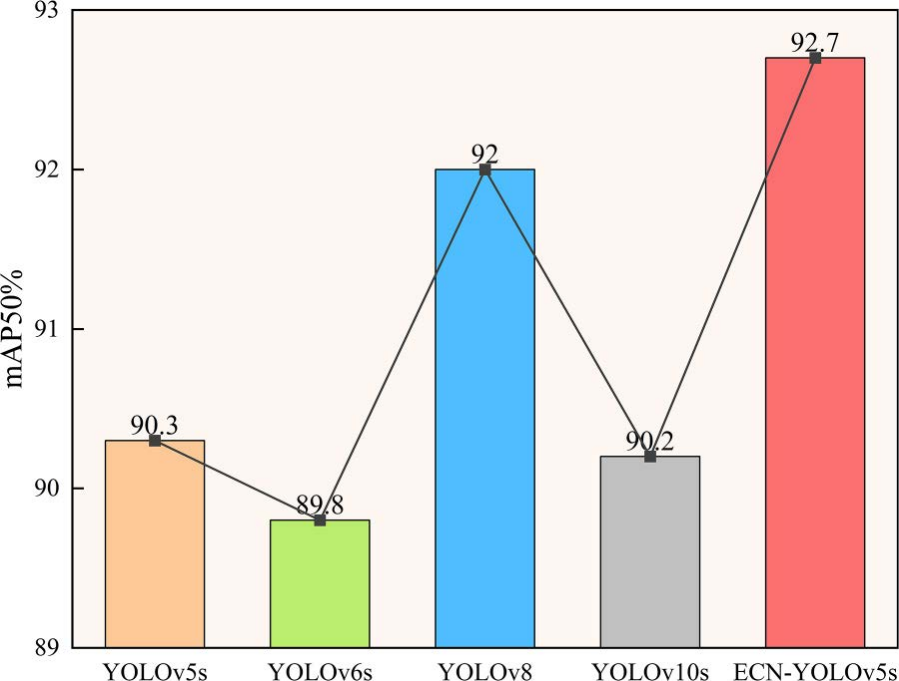
Comparison of average precision means of different algorithms.

To prove the advantages of the improved algorithm in this article, under the condition of ensuring the consistency of the training model parameters. Several mainstream algorithms of the YOLO series are used to recognize and detect the homemade bearing defect dataset and contrasted with the improved algorithm. The results of the experiment are shown in Table 6 and Fig 11. These results show that, when compared to other algorithms, the enhanced approach in this study gets the greatest mAP50 result, reaching 92.7%. It has the best effect on defect category identification. Although the FPS is not as good as YOLOv8 and YOLOv10, it is still stronger than the original model and YOLOv6, and it is capable of satisfying real-time detection demands.

**Table 6:** Performance comparison of different mainstream algorithms.

Model	Parameters (10^6^M)	FLOPs(G)	mAP50%	FPS
YOLOv5s	7.02	15.8	90.3	117
YOLOv6s	18.5	45.2	89.8	110
YOLOv8s	11.13	28.4	92	200
YOLOv10s	8.04	28.6	90.2	189
ECN-YOLOv5s	7.36	16.6	92.7	120

### Model visualization and analysis

In order to explore more intuitively and deeply the influence of CARAFE up-sampling on the aggregation effect of model contextual information and its enhancement of defect localization accuracy at different defect scales, this paper adopts the feature heatmap visualization method. The heatmap can intuitively reflect the extent of the model’s attention to the input features at different locations, and by analyzing the distribution and intensity of the activated regions in the heatmap, it can provide an in-depth understanding of the model’s ability to capture the defective regions as well as the utilization of contextual information. In this paper, Grad-CAM (Gradient-weighted Class Activation Mapping) algorithm is used to generate feature heat maps.The Grad-CAM algorithm calculates the gradient of the target class on the feature maps and performs a weighted summation of them, so as to obtain heat maps that can reflect the extent of the model’s attention to different regions of the input image.

**Fig 12:**
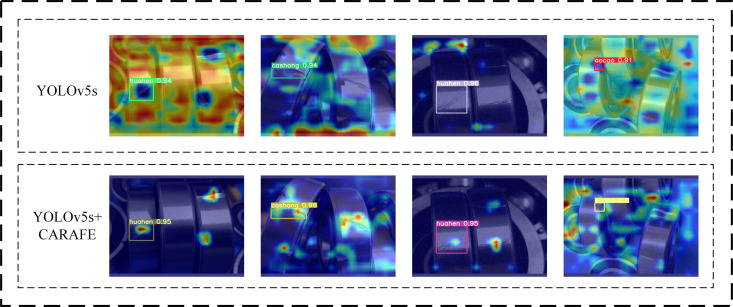
Heat maps for different models.

As shown in the Fig 12, from the generated feature heat map under the ordinary up-sampling method, it can be seen that for the small target defects in the bearing, the activation response of the model in the defective region is relatively weak, and the activation region is more dispersed, and there is a certain degree of background noise interference. In contrast, the feature heat maps generated using the CARAFE up-sampling method show stronger activation response in the defective region, and the activation region is more concentrated and accurately covers the defective region. Meanwhile, the activation response of the background region is significantly reduced, indicating that CARAFE can better suppress the background noise and highlight the defect features. This indicates that the CARAFE up-sampling method has significant advantages in aggregating contextual information and improving defect localization accuracy. Especially in the small-target defect detection task, CARAFE is able to effectively expand the receptive field, capture more contextual information that is helpful for defect localization, make the model more accurately focus on the defective region, and suppress the background noise interference. This result further validates the effectiveness and superiority of the CARAFE up-sampling method in this article.

### Ablation experiment

**Fig 13:**
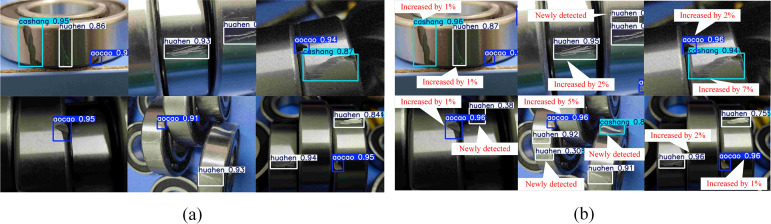
(a) YOLOv5s detection results; (b) ECN-YOLOv5s detection results. aocao stands for dent defects; cashang stands for bruise defects; huahen stands for scratch defects.

In this paper, three improvements to the YOLOv5s algorithm are proposed, and ablation experiments are conducted in order to explore the effect of each modification on the model detection precision results. Table 7 displays the findings of the experiment. Additionally, the YOLOv5s model and the modified model in this study are compared for detection in a randomly chosen dataset. The detection results are plotted in Fig 13.

**Table 7:** Results of ablation experiments.

Model	ECA	CARAFE	NWD	P(%)	R(%)	mAP50%	FPS
YOLOv5s				92.8	90	90.3	117
YOLOv5s-A	√			93.3	90.8	91.2	**139**
YOLOv5s-B		√		93.8	91	91.1	132
YOLOv5s-C			√	93.8	90.5	91.7	137
YOLOv5s-D	√	√		**94.3**	92.2	92.6	125
ECN-YOLOv5s	√	√	√	94.1	**93.1**	**92.7**	120

√ indicates the introduction of the module. YOLOv5s-A denotes that the module ECA was added to the base model; YOLOv5s-B indicates that the module CARAFE was added to the base model; YOLOv5s-C represents that the loss function was changed to NWD on the base model; YOLOv5s-D denotes that both the module ECA and the module CARAFE were added to the base model; ECN- YOLOv5s denotes the final improved model in this paper. The highest value in each column is bolded to represent optimal model performance.

By comparing with the initial model YOLOv5s prior to improvement, the following conclusions may be made:

(1) When the ECA attention mechanism was used alone, the mean defect recognition average accuracy was slightly improved. The improvement in mAP50 results was 0.9%. Furthermore, the ability to detect rate was increased by 18.8% over the initial network. It is evident that the inclusion of the ECA attention mechanism enhances detection rate and accuracy.
(2) When the up-sampling algorithm was improved for CARAFE alone, the mean defect recognition average accuracy mean was improved by 0.8% over the original YOLOv5s recognition average accuracy mean. Detection speed was improved by 12.8%. This improvement effectively optimizes the algorithm.
(3) When the NWD loss function was used alone, it can be seen that the mAP50 value was enhanced by 1.4% and the FPS value by 17.1%. It indicates that this loss function effectively improves the performance of the network.
(4) The CARAFE upsampling algorithm is improved while incorporating the ECA attention mechanism. It can be seen that although the rate of detection was only marginally increased, the mAP50 value was increased by 2.3%, and the accuracy was the highest of the several improvements. The experimental findings demonstrate that this new algorithm is significantly more effective for defect detection.
(5) Finally, this paper integrates the three improvements together. The ECN-YOLOv5s achieves the best effect on defect recognition in the experiments, with a mAP value of 92.7%, which is a 2.4% improvement compared to YOLOv5s. The recall rate is also the highest. Even though there is only a small increase in FPS, it still enhances the real-time capability of the algorithm. And as can be seen from Fig 13, the algorithm has high sensitivity to shallow small targets and hidden small targets. The results of the experiments indicate that the improved algorithm suggested in this article has a high detection performance in the identification of bearing flaws. The detection accuracy is greatly increased, which evidences the feasibility of the method improvement in this study.

### Cross-dataset testing

**Fig 14:**
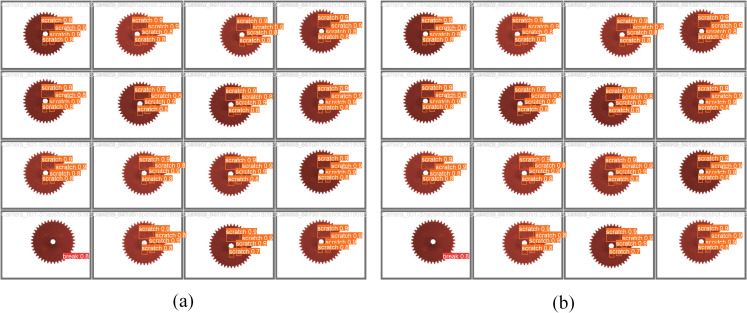
Comparison of detection results of gear defects. (a) YOLOv5s model detection results; (b) ECN-YOLOv5s model detection results.

To comprehensively evaluate the universality and generalization ability of the improved model, the gear defect dataset related to the application field of this paper was selected to test the model, in order to verify the performance of the model in different scenarios. This dataset contains three defect categories: break, lack and scratch. The break defect is usually manifested as a complete local fracture of the gear, which may present obvious fracture surfaces and irregular shapes on the image. The lack defect is the absence of material in the gear part, and the image will show cavities or depressions in the missing area. scratch defects are manifested as scratches on the gear surface, characterized by slender line-like traces. The dataset contains a total of 2,978 images, which are divided into the training set, the validation set and the test set in a ratio of 8:1:1. During the testing process, we will maintain the consistency of model parameters and training strategies in order to compare the performance differences on the two datasets more accurately. The same YOLOv5s and ECN-YOLOv5s were adopted to test this dataset, and the test results are shown in Fig 14. From the visual presentation of the detection results, we can observe intuitively and clearly that the detection effect of the improved ECN-YOLOv5s model in this paper on the gear defect dataset is superior to that of the YOLOv5s model.

The cross-dataset test results validate the generalization ability of the ECN-YOLOv5 model on different datasets. The improved model not only outperforms the baseline model in quantitative metrics, but also shows greater adaptability and robustness to different types of defects in qualitative analysis. These findings provide valuable insights for future model optimization.

## Conclusions

In this paper, an improved YOLOv5s small target detection method for bearing surface defects is proposed to realize the detection of tiny defects on the bearing surface. In order to realize the lightweight and efficient models, an efficient attention mechanism ECA is used to the backbone network. In order to give the model better diversity and robustness, and to make it adaptable to various detection circumstances and instances, the original nearest-neighbor upsampling module is substituted with CARAFE. The loss function is modified to NWD in order to increase the efficacy of overlapping tiny object detection. Extensive experiments in this study show that the ECN-YOLOv5s model mAP value is improved by 2.4% in comparison to the YOLOv5s model. The detection speed is also slightly improved. It can be concluded that the model performs better compared to the original model. It is worth thinking that this paper only investigates the model’s recognition of bearing defects, future research directions can include the recognition of defects in other parts of the machinery manufacturing industry. The overall capability and adaptability of the model can be improved by constructing more part defect datasets.

## References

[1] Dykha O, Makovkin O, Posonsky S. Influence of lubrication on the friction and wear of car rolling bearings. PT. 2021;101(3):81–8. doi: 10.31891/2079-1372-2021-101-3-81-88

[2] Liu H, Zhang Y, Li C, Li Z. Nonlinear dynamic analysis of CNC lathe spindle-bearing system considering thermal effect. Nonlinear Dyn. 2021;105(1):131–66. doi: 10.1007/s11071-021-06613-x

[3] Rejith R, Kesavan D, Chakravarthy P, Narayana Murty SVS. Bearings for aerospace applications. Tribology International. 2023;181:108312. doi: 10.1016/j.triboint.2023.108312

[4] Yu F, Chen X, Xu H, Dong H, Weng Y, Cao W. Current status of metallurgical quality and fatigue performance of rolling bearing steel and development direction of high-end bearing steel. Acta Metall Sin. 2020;56(4):513–22.

[5] Espenhahn T, Hühne R. A fully superconducting magnetic bearing with a jointless coil excited by a superconducting dynamo. Supercond Sci Technol. 2024;37(9):095021. doi: 10.1088/1361-6668/ad6d9d

[6] Yadav E, Chawla VK. An explicit literature review on bearing materials and their defect detection techniques. Materials Today: Proceedings. 2022;50:1637–43. doi: 10.1016/j.matpr.2021.09.132

[7] Ignatiev A, Dobryakov B, Ignatiev C, Kazinsky A, Nasad T, Nasad I. Automated measurements in process monitoring system in bearing production. In: Journal of Physics: Conference Series. IOP Publishing; 2020.

[8] Liang F, Zhou Y, Chen X, Liu F, Zhang C, Wu X. Review of target detection technology based on deep learning. In: 2021.

[9] Deng J, Xuan X, Wang W, Li Z, Yao H, Wang Z. A review of research on object detection based on deep learning. In: Journal of Physics: Conference Series. IOP; 2020.

[10] Bai R, Noman K, Feng K, Peng Z, Li Y. A two-phase-based deep neural network for simultaneous health monitoring and prediction of rolling bearings. Reliability Engineering & System Safety. 2023;238:109428.

[11] Tagasovska N, Lopez-Paz D. Single-model uncertainties for deep learning. Advances in Neural Information Processing Systems. 2019;32.

[12] Jiang P, Ergu D, Liu F, Cai Y, Ma B. A Review of Yolo Algorithm Developments. Procedia Computer Science. 2022;199:1066–73. doi: 10.1016/j.procs.2022.01.135

[13] Lei L, Sun S, Zhang Y, Liu H, Xie H. Segmented Embedded Rapid Defect Detection Method for Bearing Surface Defects. Machines. 2021;9(2):40. doi: 10.3390/machines9020040

[14] Kankar PK, Sharma SC, Harsha SP. Fault diagnosis of ball bearings using machine learning methods. Expert Systems with Applications. 2011;38(3):1876–86. doi: 10.1016/j.eswa.2010.07.119

[15] Gu Z, Liu X, Wei L. A detection and identification method based on machine vision for bearing surface defects. 2021 International Conference on Computer, Control and Robotics (ICCCR). IEEE; 2021.

[16] Kim DW, Lee ES, Jang WK, Kim BH, Seo YH. Effect of data preprocessing methods and hyperparameters on accuracy of ball bearing fault detection based on deep learning. Advances in Mechanical Engineering. 2022;14(2). doi: 10.1177/16878132221078494

[17] Zheng Z, Zhao J, Li Y. Research on detecting bearing-cover defects based on improved YOLOv3. IEEE Access. 2021;9:10304–15.

[18] Bapir A, Aydin İ. A comparative analysis of 1D convolutional neural networks for bearing fault diagnosis. IEEE; 2022.

[19] Udmale SS, Singh SK, Bhirud SG. A bearing data analysis based on kurtogram and deep learning sequence models. Measurement. 2019;145:665–77. doi: 10.1016/j.measurement.2019.05.039

[20] Jiang Y, Tang C, Zhang X, Jiao W, Li G, Huang T. A Novel Rolling Bearing Defect Detection Method Based on Bispectrum Analysis and Cloud Model-Improved EEMD. IEEE Access. 2020;8:24323–33. doi: 10.1109/access.2020.2970813

[21] Redmon J, Divvala S, Girshick R, Farhadi A. You only look once: unified, real-time object detection. In: Proceedings of the IEEE Conference on Computer Vision and Pattern Recognition, 2016.

[22] Kou X, Liu S, Cheng K, Qian Y. Development of a YOLO-V3-based model for detecting defects on steel strip surface. Measurement. 2021;182:109454. doi: 10.1016/j.measurement.2021.109454

[23] Li W, Zhang H, Wang G, Xiong G, Zhao M, Li G, et al. Deep learning based online metallic surface defect detection method for wire and arc additive manufacturing. Robotics and Computer-Integrated Manufacturing. 2023;80:102470. doi: 10.1016/j.rcim.2022.102470

[24] Zhou F, Chao Y, Wang C, Zhang X, Li H, Song X. A small sample nonstandard gear surface defect detection method. Measurement. 2023;221:113472. doi: 10.1016/j.measurement.2023.113472

[25] Shuai Y, Lin Z, Chen W, Shenghuai W, Yu T. SF-YOLO: An Evolutionary Deep Neural Network for Gear End Surface Defect Detection. IEEE Sensors J. 2024;24(13):21762–75. doi: 10.1109/jsen.2024.3403870

[26] Qu Z, Gao L, Wang S, Yin H, Yi T. An improved YOLOv5 method for large objects detection with multi-scale feature cross-layer fusion network. Image and Vision Computing. 2022;125:104518. doi: 10.1016/j.imavis.2022.104518

[27] Xiao D, Xie FT, Gao Y, Li ZN, Xie HF. A detection method of spangle defects on zinc-coated steel surfaces based on improved YOLO-v5. Int J Adv Manuf Technol. 2023;128(1–2):937–51. doi: 10.1007/s00170-023-11963-4

[28] Wang Q, Wu B, Zhu P, Li P, Zuo W, Hu Q. ECA-Net: Efficient channel attention for deep convolutional neural networks. In: Proceedings of the IEEE/CVF conference on computer vision and pattern recognition, 2020.

[29] Hu J, Shen L, Sun G. Squeeze-and-excitation networks. In: Proceedings of the IEEE Conference on Computer Vision and Pattern Recognition, 2018.

[30] Wang J, Chen K, Xu R, Liu Z, Loy CC, Lin D. Carafe: Content-aware reassembly of features. In: Proceedings of the IEEE/CVF International Conference on Computer Vision, 2019.

[31] Zheng Z, Wang P, Liu W, Li J, Ye R, Ren D. Distance-IoU loss: faster and better learning for bounding box regression. In: Proceedings of the AAAI Conference on Artificial Intelligence, 2020.

[32] Wang J, Xu C, Yang W, Yu L. A normalized Gaussian Wasserstein distance for tiny object detection. 2021. https://arxiv.org/abs/2110.13389

